# Sensitization effect of kaempferol from persimmon leaves on HepG2 hepatoma cells with ABT-199 resistance and its molecular mechanisms

**DOI:** 10.3389/fphar.2022.1032069

**Published:** 2022-11-01

**Authors:** Li Chen, Xudong Jiang, Si Gao, Xueping Liu, Ying Gao, Audrey Siew Foong Kow, Chau Ling Tham, Ming Tatt Lee

**Affiliations:** ^1^ Faculty of Pharmaceutical Sciences, UCSI University, Kuala Lumpur, Malaysia; ^2^ Department of Pharmacology, College of Medicine, Guangxi University of Science and Technology, Liuzhou, China; ^3^ International Ginseng Institute, School of Agriculture, Middle Tennessee State University, Murfreesboro, TN, United States; ^4^ Department of Biomedical Sciences, Faculty of Medicine and Health Sciences, Universiti Putra Malaysia, Serdang, Selangor, Malaysia

**Keywords:** hepatocarcinoma, preclinical model, kaempferol, persimmons leaves, ABT-199, Bcl-2, Mcl-1, chemoresistance

## Abstract

ABT-199 (venetoclax) is the first-in-class selective B-cell lymphoma 2 (BCL2) inhibitor, which is known to be ineffective towards liver cancer cells. Here, we investigated the efficacy and the underlying molecular processes of the sensitization effect of kaempferol isolated from persimmon leaves (KPL) on the ABT-199-resistant HepG2 cells. The effects of various doses of KPL coupled with ABT-199 on the proliferation of HepG2 cells and on the H22 liver tumor-bearing mouse model were examined, as well as the underlying mechanisms. Our findings showed that ABT-199 alone, in contrast to KPL, had no significant impact on hepatoma cell growth, both *in vitro* and *in vivo*. Interestingly, the combination therapy showed significantly higher anti-hepatoma efficacy. Mechanistic studies revealed that combining KPL and ABT-199 may promote both early and late apoptosis, as well as decrease the mitochondrial membrane potential in HepG2 cells. Western blot analysis showed that combination of KPL and ABT-199 significantly reduced the expression of the anti-apoptotic proteins Bcl-2, Bcl-xL, and Mcl-1, raised the expression of Bax and cleaved caspase 3, and enhanced cytochrome C release and Bax translocation. Therefore, KPL combined with ABT-199 has a potential application prospect in the treatment of hepatocellular carcinoma.

## 1 Introduction

Hepatocellular carcinoma (HCC), the most common type of primary liver cancer, is one of the most well-known harmful tumours. The death rate is second to gastric cancer and cellular breakdown in the lungs and is the fifth driving reason for tumour-related deaths in the world (Siegel et al., 2018). Most patients with HCC rely on chemotherapy and radiotherapy for treatment, but conventional chemotherapy and radiotherapy often fail to achieve satisfactory results (Liu et al., 2015). Although surgical removal can be regarded as curative treatment therapy, recurrence remains as a major issue (Qian et al., 2021). Furthermore, liver cancer is often diagnosed at advanced stages, which surgical intervention may not be feasible. Therefore, finding new therapeutic drugs and therapeutic targets has become an urgent task for medical researchers.

ABT-199 (Venetoclax) is the first Bcl-2 inhibitor approved by FDA for the treatment of patients with chronic lymphocytic leukemia, small lymphocytic lymphoma (SLL) and acute myeloid leukemia (AML) (Cang et al., 2015; Deeks, 2016). Several reports on the therapeutic potentials of ABT-199 on chronic lymphocytic leukemia (Souers et al., 2013), breast cancer (Vaillant et al., 2013) and other malignant hematologic cancers and solid tumours (Itchaki and Brown, 2016; Shi et al., 2021; Weller et al., 2022). To the best of our knowledge, there’s no reports to date demonstrating the effects of ABT-199 in HCC. Many tumour cells over-express Mcl-1 protein, resulting in the imbalance of interaction between anti-apoptotic members and pro-apoptotic members, leading to malignant proliferation of tumour cells. Studies have reported that, as an important anti-apoptotic protein, Mcl-1 is also over-expressed in hepatocellular carcinoma (Fleischer et al., 2006), which is one of the important factors of HCC drug resistance (Sieghart et al., 2006). The rational for the combination of agents based on the mechanism of antineoplastic drugs and the dynamics of proliferation is gaining attention in the field of tumour chemotherapy in recent years, and it is also one of the effective methods to overcome tumour resistance (Boshuizen and Peeper, 2020).

Persimmon leaves are the fresh or dried leaves of *Diospyros kaki* L. f., a species of the Ebenaceae family. Previous literature demonstrated that persimmon leaves have many ethnopharmacological properties, such as reducing blood pressure, hemostasis, reducing blood sugar, reducing blood lipid, antioxidant, antibacterial, liver protection, anti-tumour, etc (Kim et al., 2020a; Kim et al., 2020b). In our previous studies, we found that the ethyl acetate part of persimmon leaves and the total flavones of persimmon leaves could inhibit the tumour growth of H22 tumour-bearing mice (Chen et al., 2018). We also showed that the total flavones extracted from persimmon leaves had significant inhibitory effects on liver cancer and breast cancer, by regulating the redox state and increasing the level of ROS in cells to promote apoptosis (Chen et al., 2020). The main active components of total flavonoids in persimmon leaves are kaempferol, quercetin and its glycosides (Liu et al., 2012; Xie et al., 2015). Among these flavonoids, several studies have confirmed that kaempferol has broad-spectrum anti-tumour activities against liver cancer, breast cancer, colon cancer, prostate cancer, bladder cancer, cervical cancer, etc (Wang et al., 2019; Zhang et al., 2022). Epidemiological studies showed that dietary intake of flavonoids, including kaempferol, is correlated with low incidences of liver cancer (Zamora-Ros et al., 2013). Previous molecular studies have shown that kaempferol inhibits HepG2 cell proliferation, migration, and invasion, as well as cell viability in a dose- and time-dependent manner (Zhu et al., 2018). Furthermore, treatment with kaempferol can induce apoptosis and cause cell cycle arrest at the G2/M phase, thus preventing tumour cell migration and invasion (Huang et al., 2013; Sharma et al., 2021). Interestingly, previous research has linked kaempferol’s anti-tumour effect to Bcl-2 family proteins that regulate the endogenous mitochondrial apoptosis pathway, which can downregulate Bcl-2 and Mcl-1 while increasing Bax protein expression (Imran et al., 2019; Zhu and Xue, 2019; Afroze et al., 2022). Therefore, in this study, we hypothesized that KPL can increase the sensitivity of hepatocarcinoma cells that is resistant to ABT-199, by down-regulating the expression of Mcl-1. Furthermore, we examined the sensitization effect and elucidated the underlying molecular mechanism of the selective Bcl-2 inhibitor ABT-199 combined with KPL on HepG2 cell lines *in vitro* and *in vivo*.

## 2 Materials and methods

### 2.1 Cell-line and animals

HepG2 cells were purchased from Stem Cell Bank, Chinese Academy of Sciences (SCB, Shanghai, China), and were routinely cultured according to the protocol provided by SCB.

H22 ascites tumour mice were obtained from Guangxi Institute of Traditional Chinese Medicine and Pharmaceutical Science. Six-week-old Kunming mice (male and female, SPF) were purchased from Human SJA Laboratory Animal Co., Ltd., weighing at 18–22 g (Certificate No. SYXK 2019–0017, Changsha, China). All animal experimentations reported in this study were carried out in accordance with the guidelines for the use and care of experimental animals and were approved by the Experimental Animals Committee at Guangxi University of Science and Technology. The mice were housed at room temperature between 20–26°C, the humidity was 40–70%, under a 14:10 light/dark cycle. The mice had free access to feed and drinking water for the whole experimentation period.

### 2.2 Chemicals and reagents

Silica gel column chromatography was purchased from Marine Chemical plant (Qingdao, China). Petroleum ether 60, ethyl acetate, chloroform, methanol and glacial acetic acid were purchased from Xilong Chemical Co., Ltd. (Guangzhou, China). Dimethyl Sulfoxide (DMSO) was purchased from Kulaibo Technology Co., Ltd. (Beijing, China). Eagle’s minimum essential medium (EMEM), trypsin, and fetal bovine serum (FBS) were purchased from Shuangru Biotechnology Co., Ltd. (Shanghai, China). Persimmon (*Diospyros* kaki L.) leaves were purchased from Yaoyuan Trading Inc. (Anguo City, Hebei Province, China) (Batch No. 161011). ABT-199 and kaempferol reference standards were purchased from TargetMol (MA, United States).

Cell counting kit-8 (CCK-8), RIPA lysis buffer, PMSF solution, Quick block solution, primary antibody dilution buffer, secondary antibody dilution buffer, SDS-PAGE gel preparation kit, Tris buffered saline, SDS-PAGE electrophoresis buffer, pre-stained color protein ladder, cell mitochondria isolation kit, 4% paraformaldehyde fix solution and crystal violet staining solution were purchased from Beyotime (Shanghai, China). Hoechst 33,342, Annexin V, and propidium iodide (PI) were purchased from BD BioSciences (San Jose, CA, United States). JC-1 mitochondrial membrane potential assay kit was purchased from Cayman Chemical (MI, United States). Polyvinylidene difluoride (PVDF) membrane and ECL reagent were purchased from Millipore (MA, United States). BCA protein assay kit was purchased from Merck (novagen series, Germany). The manufactures and catalogue numbers of antibodies used in this study are listed as follows, Bcl-2 (Cell Signaling Technology, MA, United States, #4223T, 1:1000), Bcl-xL (Cell Signaling Technology, MA, United States, # 2764T, 1:1000), Bax (Cell Signaling Technology, MA, United States, #5023T, 1:1000), Mcl-1 (Cell Signaling Technology, MA, United States, #5453T, 1:1000), Caspase 3 (Cell Signaling Technology, MA, United States, #9665T, 1:1000), α-Tubulin (Cell Signaling Technology, MA, United States, #2125S, 1:1000), Cyt C (Cell Signaling Technology, MA, United States, #4280T, 1:1000), secondary antibody (horseradish peroxidase-conjugated goat anti-rabbit) (Cell Signaling Technology, MA, United States, sub-packaged by Asbio Technology, # as006, 1:1000).

### 2.3 Kaempferol isolated from persimmon leaves (KPL) and the content determination

Kaempferol from persimmon leaves (KPL) was isolated from the total flavonoids of persimmon leaves by the Pharmacological Laboratory of Pharmacy, Department of Medicine, Guangxi University of Science and Technology. The extraction method was described by Chen et al. previous studies (Chen et al., 2018; Chen et al., 2020). Briefly, 20.0 g of persimmon leaves was separated by silica gel column chromatography (360.0 g, 200∼300 mesh sieves). The crude powder was obtained by gradient elution of petroleum ether 60 - ethyl acetate (100:0 → 100:1 → 80:1 → 50:1 → 20:1 → 10:1 → 5:1 → 2:1 → 0:100, V/V). The compound in powder form (12.4 mg) was obtained by repeated silica gel column chromatography with chloroform - methanol - glacial acetic acid (75:15:1, V/V) as eluent. The content of KPL was determined by HPLC, and the standard curve was prepared with different concentrations of kaempferol reference materials. Chromatographic conditions: the chromatographic column is Ultimate XB-C18 (5 μm, 4.6 mm × 250 mm); the mobile phase was acetonitrile: 0.2% phosphoric acid aqueous solution = 32: 68; the flow rate was 1.0 ml/min; the detection wavelength was 360 nm; the column temperature was room temperature; the sample volume was 20 μL.

### 2.4 Cell culture

HepG2 cell line used in this study was cultured in EMEM medium, supplemented with 10% FBS, 100 U/ml of penicillin and 100 U/ml of streptomycin at a 37°C incubator (Thermo, United States) supplied with 5% CO_2_. The cells in the logarithmic growth phase were applied in the following assays.

### 2.5 Cell growth inhibition assays of ABT-199

Cells (5×10^3^ cells/well) were seeded in 96-well plates, cultured overnight to allow cells attachment, and treated with ABT-199 at the concentrations of 1, 2, 5, 10 and 15 μM respectively. After 48 h treatment, 10 μL of CCK-8 dye was added into each well to a final concentration (v/v) of 10% and incubated for another 2 h. Subsequently, the absorbance (OD) was measured at 450 nm by Multiskan GO microplate reader of multi-wavelength measurement system (Thermo Scientific, MA, United States). Cell growth inhibition rate was evaluated as the ratio of the absorbance of the treated samples to that of the negative control samples and analyzed by Prism 6 software (GraphPad Software Inc. San Diego, CA, United States). All experiments were carried out in triplicate. The formula of cell growth inhibition (%) is as follows:

$$growth\;inhibition\;\left(\%\right)=\left(1-\frac{ODT}{ODC}\right)\times 100\%$$

Where ODT is the average OD value of the treated samples and ODC is the average OD value of the negative control samples.

### 2.6 Cell growth inhibition assays of ABT-199 combined with KPL

**FIGURE 1:**
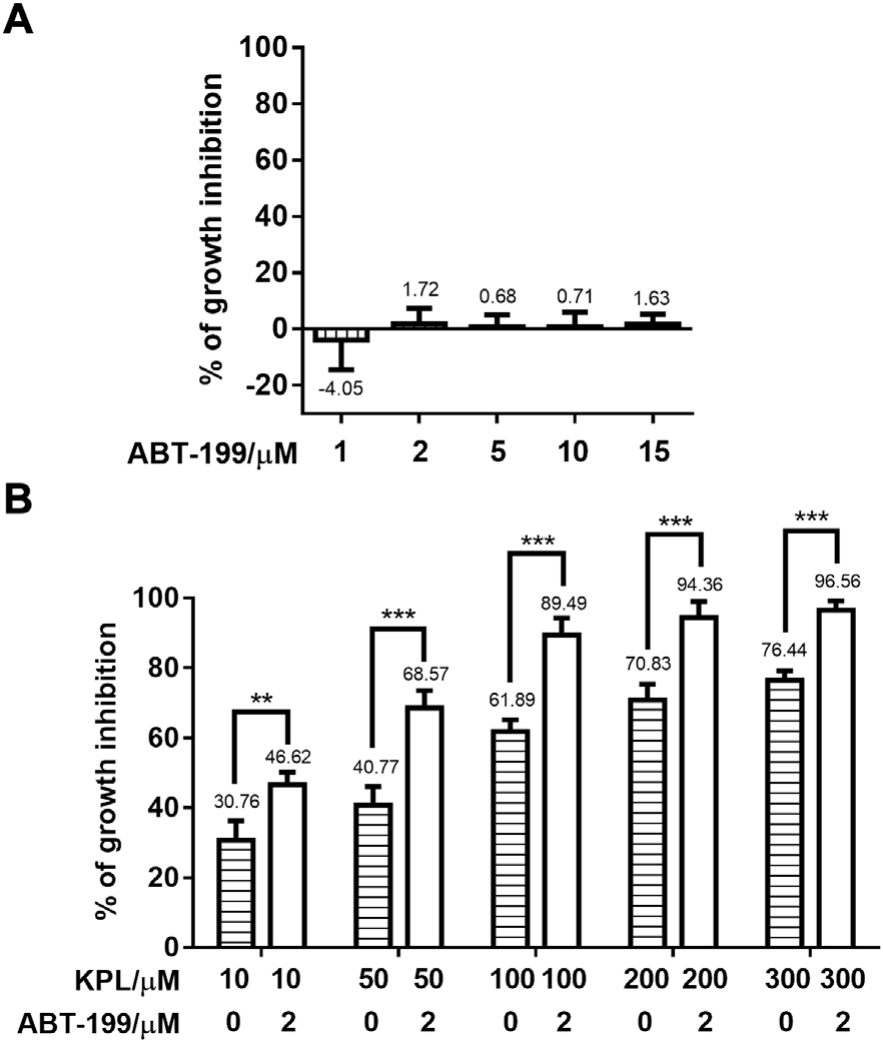
Effect of KPL combined with ABT-199 on the growth inhibition of HepG2 cells. **(A)** Growth inhibition percentage (%) of different concentrations of ABT-199 against HepG2 cell line. Cells were seeded in a 96-well plate and treated with 1–15 μM ABT-199 for 48 h, and the growth inhibition percentage of cells was detected with CCK-8 kit. **(B)** Growth inhibition percentage (%) of different concentrations of KPL combined with 2 μM ABT-199 on HepG2 cell line. Cells were seeded in a 96-well plate and treated with 10–300 μM KPL for 48 h with or without 2 μM ABT-199, and the growth inhibition percentage of cells was determined by CCK-8 kit. Values are means ± SD (*n* = 4). **p < 0.01, ***p < 0.001 *versus* the same concentration KPL only group.

According to the preliminary experimental results (Figure 1A), the growth inhibition rate (%) of ABT-199 on HepG2 cells at the above five different concentrations were all less than 2% without significant difference, indicating that ABT-199 did not inhibit the proliferation of HepG2 cells. Thus, 2 μM of ABT-199 was selected for the subsequent experiments. Cells (5 × 10^3^ cells/well) were seeded in 96-well plates, cultured overnight to allow cells attachment, and treated with KPL at the concentration of 10, 50, 100, 200 and 300 μM alone or combined with 2 μM of ABT-199 to the corresponding wells for 48 h. Cell growth inhibition assays were conducted as described in Section 2.5 (Cell growth inhibition assays of ABT-199).

### 2.7 Colony forming assay

Cells were seeded in 6-well plates at 1 × 10^5^ cells/well and incubated overnight. The cells were next treated with KPL at the concentration of 100 and 200 μM alone or combined with ABT-199 at 2 μM to the corresponding wells. After treatment for 24 h, the culture medium was discarded and fresh complete medium was added to continue the culture. The culture medium was changed every 3–4 days until there were colonies visible to the naked eye (about 14 days). After that, the culture solution was discarded, 4% paraformaldehyde was added to the wells and the cells were fixed for 10min. After fixation, the cells were washed with distilled water and stained with crystal violet staining solution for 10 min. After staining, the staining solution was removed through repeated washing. Photos were taken and the formation of cell clones in each group was compared by using Image J (NIH, United States). The experiment was carried out in quadruplicate as described previously with minor modifications (He et al., 2020).

### 2.8 Apoptosis assay

HepG2 cells were seeded in a 96-well clear bottom black plate at a density of 5 × 10^3^ cells/well and cultured overnight. Following attachment, cells were treated with KPL at the concentration of 100 and 200 μM alone or combined with ABT-199 at 2 μM to the corresponding wells for 24 h. The negative control group was treated with complete medium only. Each treatment group had four replicates. After treatment for 24 h, 1 μL Hoechst 33342, 5 μL PI, and 5 μL FITC-Annexin V were added to each well. Apoptosis in each group was evaluated and analyzed by ArrayScan VTI High Content Screening reader (Thermo Scientific, MA, United States). The Excitation/Emission wavelengths for Hoechst 33,342, FITC-Annexin V, and PI were 350/461 nm, 494/518 nm, and 535/617 nm, respectively (Alsaif et al., 2017).

### 2.9 Determination of mitochondrial membrane potential

Cells were seeded in 6-well plates at 3 × 10^5^ cells/well and incubated overnight. The cells were next treated with KPL at the concentration of 100 and 200 μM alone or combined with ABT-199 at 2 μM to the corresponding wells. After treatment for 24 h, the culture medium was discarded, and the cells were washed with PBS. Then JC-l Staining Solution with the final concentration of 10 µg/ml was added to the cells followed by incubation in the dark at 37°C for 30 min. The supernatant was discarded, and the cells were washed with assay buffer before observation. The images were observed in dark and captured under TS2-FL ECLIPSE inverted fluorescence microscope (Nikon Imaging Japan Inc.).

### 2.10 Western blot assay

The effects of KPL combined with ABT-199 on apoptosis-related proteins were detected in the total protein of HepG2 cells. Cells were seeded in 6-well plates at 5 × 10^5^ cells/well and incubated overnight. After the cells were treated with corresponding reagents for 24 h, the culture medium was discarded and the cells were washed with PBS. Then, the cells were lysed with RIPA containing protease inhibitor PMSF, and centrifuged at 1.2 × 10^4^ g for 15 min at 4°C to collect the supernatant. The effects of KPL combined with ABT-199 on Cyt C release and Bax translocation in HepG2 cells were detected in mitochondria and cytoplasmic proteins. The mitochondria proteins were isolated according to the instruction of cell mitochondria isolation kit manual (Cayman Chemical, MI, United States). All the proteins concentration was quantified by the BCA method.

Equal amounts of protein (20–40 μg) were separated by 12% SDS-PAGE gel electrophoresis and transferred onto PVDF membranes. After blocking with 5% TBST milk, the membranes were hybridized with primary antibodies: Bcl-2, Bcl-xL, Bax, Mcl-1, Caspase 3, cleaved caspase 3, Cyt C and α-Tubulin. The ratio of primary antibody and primary antibody diluent is 1:1000. All the membranes were incubated with their respective primary antibodies overnight at 4°C and the corresponding horseradish-peroxidase-conjugated secondary antibodies for 1 h at room temperature. α-Tubulin was used as the housekeeping protein. The blots were developed using ECL reagent and visualized by an automatic chemiluminescence imaging system (GelView 6000 Pro, Guangzhou bolutang Instrument Co., Ltd.), and the protein levels were quantified using Image J software.

### 2.11 Animal models and experimental design

The solid tumour model was established using the method previously described (Chen et al., 2018). In brief, four ascites tumour mice with H22 cells passaged in the abdominal cavity for 7–9 days were taken, the mice were sacrificed, and ascites were collected under sterile conditions. The ascites was diluted to 1 × 10^6^ cells/mL with normal saline and 0.2 ml of the ascites was inoculated subcutaneously to healthy Kunming mice in the armpit of the right forelimb. After 24 h inoculation, the mice were randomly divided into 4 groups with 10 mice in each group (half male and half female): control, ABT-199 (100 mg/kg), KPL (100 mg/kg), KPL (100 mg/kg) +ABT-199 (100 mg/kg). The mice were treated by intraperitoneal injection (*i.p.*) daily for 10 continuous days. On the 11th day, the mice were sacrificed by cervical dislocation, the tumour weights were measured, and the tumour inhibition rates were calculated using the following formula:

$$tumour\;inhibition\;\left(\%\right)=\frac{tumour\;weight\;in\;model\;group\;\left(g\right)-tumour\;weight\;in\;experimental\;group\;\left(g\right)}{tumour\;weight\;in\;model\;group\;\left(g\right)}\times 100\%$$

### 2.12 Statistical analysis

The experimental data were analysed by SPSS 25.0 statistical software (IBM, Chicago, IL, United States), and the measurement data were expressed as mean ± SD. One-way analysis of variance (ANONA) followed by Tukey’s *post hoc* test was used for comparison between multiple groups. *p* < 0.05 is considered as statistically significant.

## 3 Results

### 3.1 Identification and content determination of KPL

The compound, KPL, was in the form of yellow acicular crystal with the melting point of 276–278°C. KPL was soluble in chloroform, methanol, ethyl acetate and acetone but insoluble in petroleum ether and water. The compound was presented as yellow in the 10% sulfuric acid ethanol test and showed positive in the ferric chloride reaction and magnesium hydrochloride powder reaction. The ^13^C NMR data (Table 1) of KPL is consistent with the ^13^C NMR data of kaempferol reported in the previous literature (Chen et al., 2018; Chen et al., 2020). Therefore, it is concluded that the compound isolated from PLF was KPL, and the content of KPL was 91.23% as determined by HPLC.

**TABLE 1:** ^13^C NMR data of KPL (100 MHz, DMSO d6).

C position	Experimental value δc	Reference value δc	C position	Experimental value δc	Reference value δc
C-2	146.9	146.1	C-9	161.3	160.5
C-3	136.6	135.5	C-10	103.8	102.9
C-4	176.5	175.7	C-1′	122.6	121.6
C-5	157.1	156.0	C-2′, 6′	130.4	129.3
C-6	98.8	98.2	C-3′, 5′	116.4	115.3
C-7	164.7	163.8	C-4′	159.8	159.0
C-8	94.2	93.4			

### 3.2 The inhibitory effect of KPL and ABT-199 co-administration on the growth of Mcl-1 overexpressing HepG2 cell line

HepG2 cell line with high expression of Mcl-1 (Yu et al., 2016) was employed in this study. First, we examined the potential growth inhibition effect of ABT-199, ranging from 1–15 μM using CCK-8 staining. As shown in Figure 1A, the growth inhibitory effect of ABT-199 at all tested concentrations were lower than 2%, which showed that ABT-199 had no inhibitory effect against HepG2 cell line, proving that hepatoma cells were insensitive to ABT-199. ABT-199 at 2 μM was chosen as the combination treatment with KPL for the subsequent experiments. As shown in Figure 1B, KPL alone (horizontal slashed bars, 10–300 μM) significantly inhibited the growth of HepG2 cells, in a concentration-dependent manner. Interestingly, when combined with 2.0 µM of ABT-199, the inhibitory effect of KPL is significantly higher at each tested concentration, as compared with KPL alone. These results indicate that KPL may have sensitization effect on HepG2 cells for ABT-199.

### 3.3 The inhibitory effect of KPL combined with ABT-199 on colony-forming ability of HepG2 cells

**FIGURE 2:**
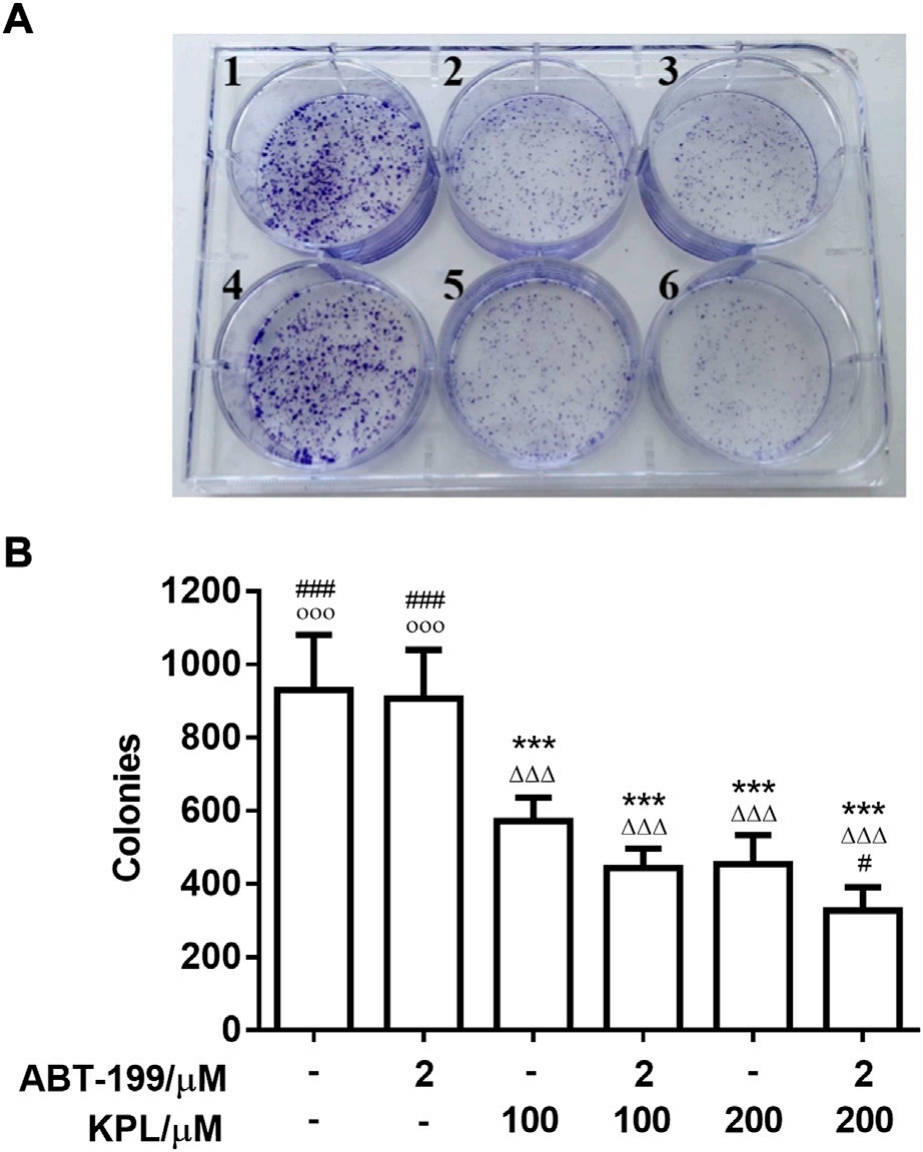
The experimental results of clone formation of HepG2 cells treated with KPL and ABT-199 for 24 h. **(A)** Colony formation assay was conducted to investigate growth *in vitro* after treatment with KPL at the concentration of 100 and 200 μM alone or combined with ABT-199 at 2 μM for 14 days. The colonies were visualized with the images. 1: Negative control, 2: KPL 100 μM, 3: KPL 200 μM, 4: ABT-199 2 μM, 5: KPL 100 μM + ABT-199 2 μM, 6: KPL 200 μM + ABT-199 2 μM. **(B)** The corresponding histogram showed the colony numbers. Values are means ± SD (*n* = 4). ***p < 0.001 *versus* negative control group; ΔΔΔ*p*<0.001 *versus* ABT-199 alone group; ^#^ *p* < 0.05, ^###^ *p* < 0.001 *versus* KPL 100 μM group; ^○○○^ *p* < 0.001 *versus* KPL 200 μM group.

To further investigate the effect of KPL combined with ABT-199 on the proliferation of HepG2 hepatoma cells, the changes of cell clone formation of HepG2 after being treated with KPL combined with ABT-199 for 24 h were detected by crystal violet staining. As shown in Figure 2A, in HepG2 cells treated with ABT-199 (2 µM) alone, the number of colonies were not significantly different from the control group, in contrast with KPL alone groups (100–200 μM). However, the number of colonies at all concentration of KPL combined with ABT-199 were significantly lower than that of the negative control group and ABT-199 alone. As shown in Figures 2A,B, the number of colonies formed was smaller and less in a concentration-dependent manner, which was consistent with the results of CCK-8 (Figure 1).

### 3.4 The effect of KPL combined with ABT-199 on tumour growth and tumour cell necrosis in H22 tumour-bearing mice

**FIGURE 3:**
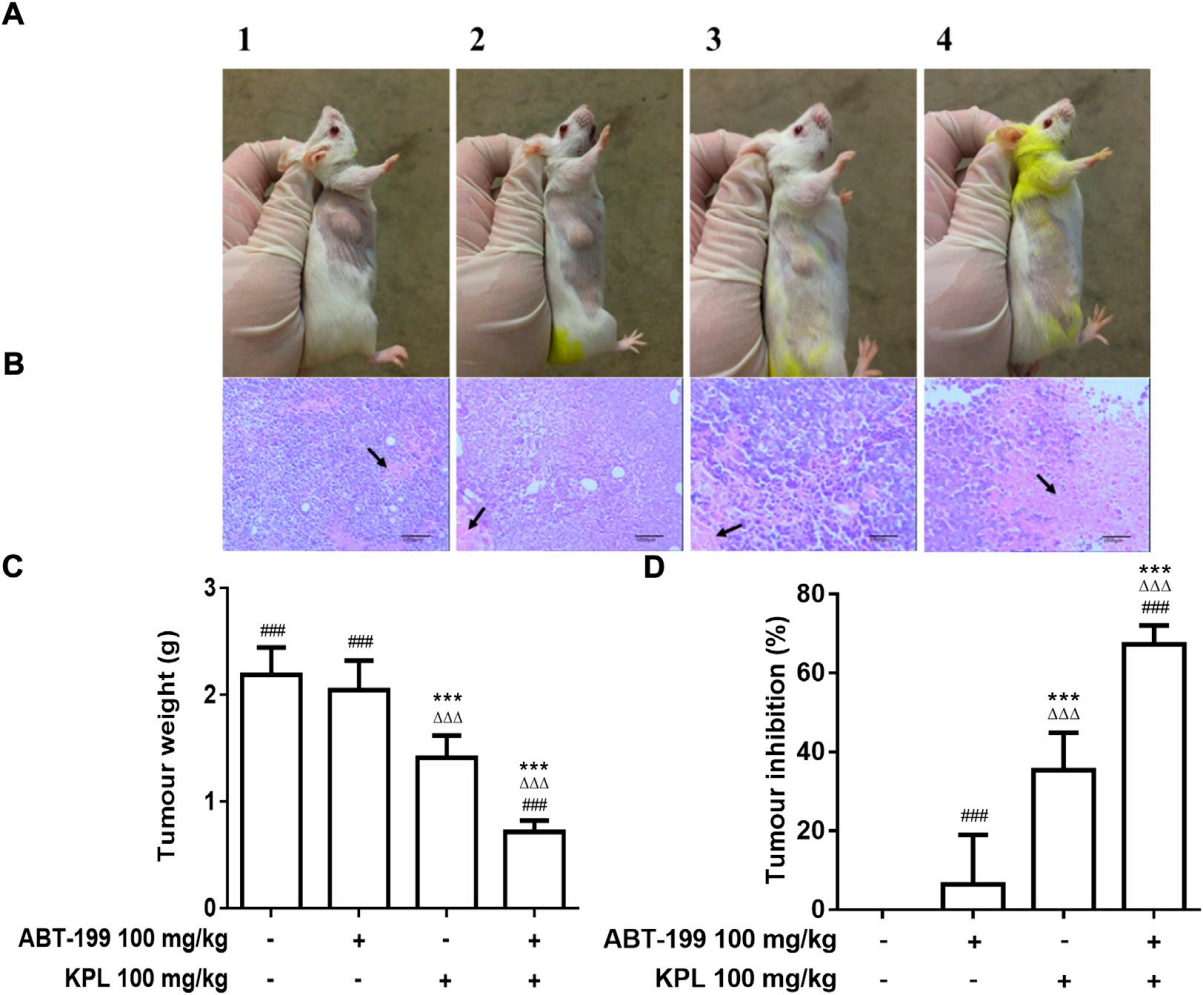
The effect of KPL combined ABT-199 on tumour growth and tumour cells necrosis in H22 tumour-bearing mice. **(A)** Representative images of the tumors in various groups. The size of tumors in each group can be seen with the naked eye: compared with the control group, the tumour size of KPL only group and KPL combined with ABT-199 group were significantly reduced, whereas the KPL combined with ABT-199 group were more significantly reduced. A1: Control group, A2: ABT-199 100 mg/kg group, A3: KPL 100 mg/kg group, A4: KPL 100 mg/kg + ABT-199 100 mg/kg group. **(B)** Histological examination of tumour tissues from H22 tumor-bearing mice. The tumorus were sectioned and stained with H&E. The necrotic tumor foci were indicated by the black arrows. B1: Control group, B2: ABT-199 100 mg/kg group, B3: KPL 100 mg/kg group, B4: KPL 100 mg/kg + ABT-199 100 mg/kg group. **(C)** Tumor weights of the mice in each group at the end of the experiment. **(D)** Tumor inhibition percentage of the various treatments. Magnification: 100 ×. Values are means ± SD (*n* = 10). ***p < 0.001 *versus* negative control group; ^ΔΔΔ^ *p* < 0.001 *versus* ABT-199 alone group; ^###^ *p* < 0.001 *versus* KPL alone group.

Next, we employed the H22 solid liver cancer-bearing mice model to determine whether systemic administration of ABT-199 combined with KPL could inhibit tumour growth in mice. The *in vivo* dose of ABT-199 (100 mg/kg, *i.p.*) was selected based on previous similar studies (Pan et al., 2014; Peirs et al., 2014). In our preliminary study, we found that treatment with 100 mg/kg and 200 mg/kg (*i.p.*) of KPL displayed similar efficacy, thus 100 mg/kg KPL was selected for *in vivo* experiment. After repeated treatment with *i.p.* ABT-199 alone or combined with KPL for 10 days, the tumour weights were measured, and the tumour inhibition percentages were calculated. As showed in Figures 3A,C, compared with the control group (2.188 ± 0.256 g), the tumour weight of ABT-199 only group (2.047 ± 0.275 g) was not significantly reduced, but the tumour weight of KPL only group (1.412 ± 0.206 g) and KPL combined with ABT-199 group (0.716 ± 0.105 g) were significantly reduced (*p* < 0.001). Noticeably, the tumour inhibition by KPL-ABT-199 co-treatment group was significantly higher than ABT-199 alone and KPL alone groups (Figures 3C,D). These results also indicate that the KPL displayed a significant sensitization effect *in vivo*, which is consistent with the *in vitro* findings in Figure 1 and Figure 2.

We also conducted a hematoxylin-eosin (H&E) pathological analysis with the tumour sections to confirm the sensitization effect of KPL *in vivo*. The H&E staining in Figure 3 demonstrated that the tumour cells in the control group were irregularly arranged, with large volume, large nucleocytoplasmic ratio and obvious atypia. The morphological characteristics of tumour cells in ABT-199 only group were the same as those in the control group; In KPL only group, there were flake necrosis and less pathological mitosis in tumour tissue; In ABT-199 combined with KPL group, the tumour sections exhibited notably different histological features, obvious flake coagulation necrosis was observed in the tumour tissue, the necrosis area were significantly larger than that in other groups, the tumour nucleus were pyknosis, and the necrotic cells were mainly located between loosely arranged cells. The experimental results showed that KPL could enhance the anti-hepatoma effect of ABT-199.

### 3.5 The effect of KPL combined with ABT-199 on apoptosis of HepG2 cells

**FIGURE 4:**
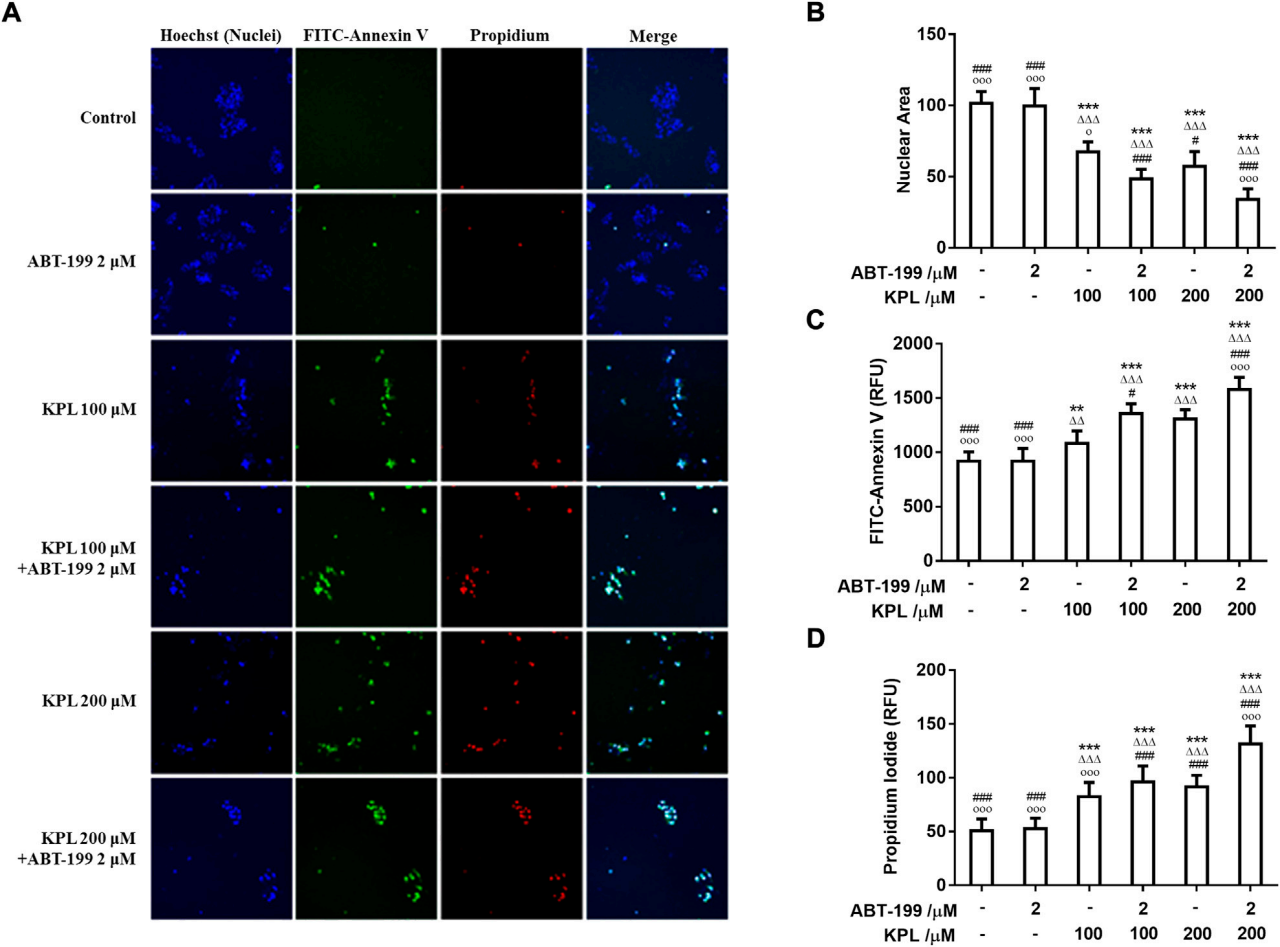
The effect of KPL combined with ABT-199 on apoptosis of HepG2 cells. **(A)** Representative images of cell apoptosis in various groups. Different concentrations of KPL with or without 2 μM ABT-199 were treated on HepG2 cells for 24 h, and then the apoptosis was detected by Hoechst 33342/FITC-Annexin V/PI triple staining. The images were collected by an Arrayscan VT1 HCS reader. Merged images were the composition of images of the same field from three different channels. **(B)** The effects of various concentrations of KPL with or without ABT-199 on the nuclear area. **(C)** Fluorescence intensity of FITC Annexin V staining in HepG2 cells treated by various concentrations of KPL with or without ABT-199. **(D)** Fluorescence intensity of PI staining in HepG2 cells treated by various concentrations of KPL with or without ABT-199. Values are means ± SD (*n* = 12). **p < 0.01, ***p < 0.001 *versus* negative control group; ^ΔΔ^ *p* < 0.01, ^ΔΔΔ^ *p*<0.001 *versus* ABT-199 alone group; ^#^ *p* < 0.05, ^###^ *p* < 0.001 *versus* KPL 100 μM group; ^○^ *p* < 0.05, ^○○○^ *p* < 0.001 *versus* KPL 200 μM group.

In this study, we investigated whether the inhibitory effect of KPL combined with ABT-199 can be related to the induction of apoptosis. We utilised the Hoechst 33342/FITC-Annexin V/PI triple staining assay to detect apoptosis in HepG2 cells. The findings in Figures 4A,B showed that after Hoechst 33342 staining, the nuclei of cells treated by ABT-199 appeared dark blue, similar to the negative control group, with no significant difference in nuclear area between the two groups. However, in the HepG2 cells that were treated with all concentrations of KPL and KPL combined with ABT-199, the nuclei area was significantly decreased (*p* < 0.001), and appeared bright blue, which is one of the classical hallmarks of apoptosis. As shown in Figures 4A,C,D, Annexin V and PI staining of cells in the negative control group and ABT-199 only group showed almost no fluorescence, indicating that there was almost no apoptosis, but cells treated with all concentrations of KPL, the fluorescence intensity of both FITC-Annexin V and PI was significantly increased, especially KPL combined with ABT-199 group (*p* < 0.01 or *p* < 0.001). The number of cells were less than that of KPL only group, due to the combined treatment inhibited the cells’ proliferation. The results implied that KPL combined with ABT-199 could promote the early and late apoptosis of HepG2 cells.

### 3.6 The effect of KPL combined with ABT-199 on mitochondrial membrane potential of HepG2 cells

**FIGURE 5:**
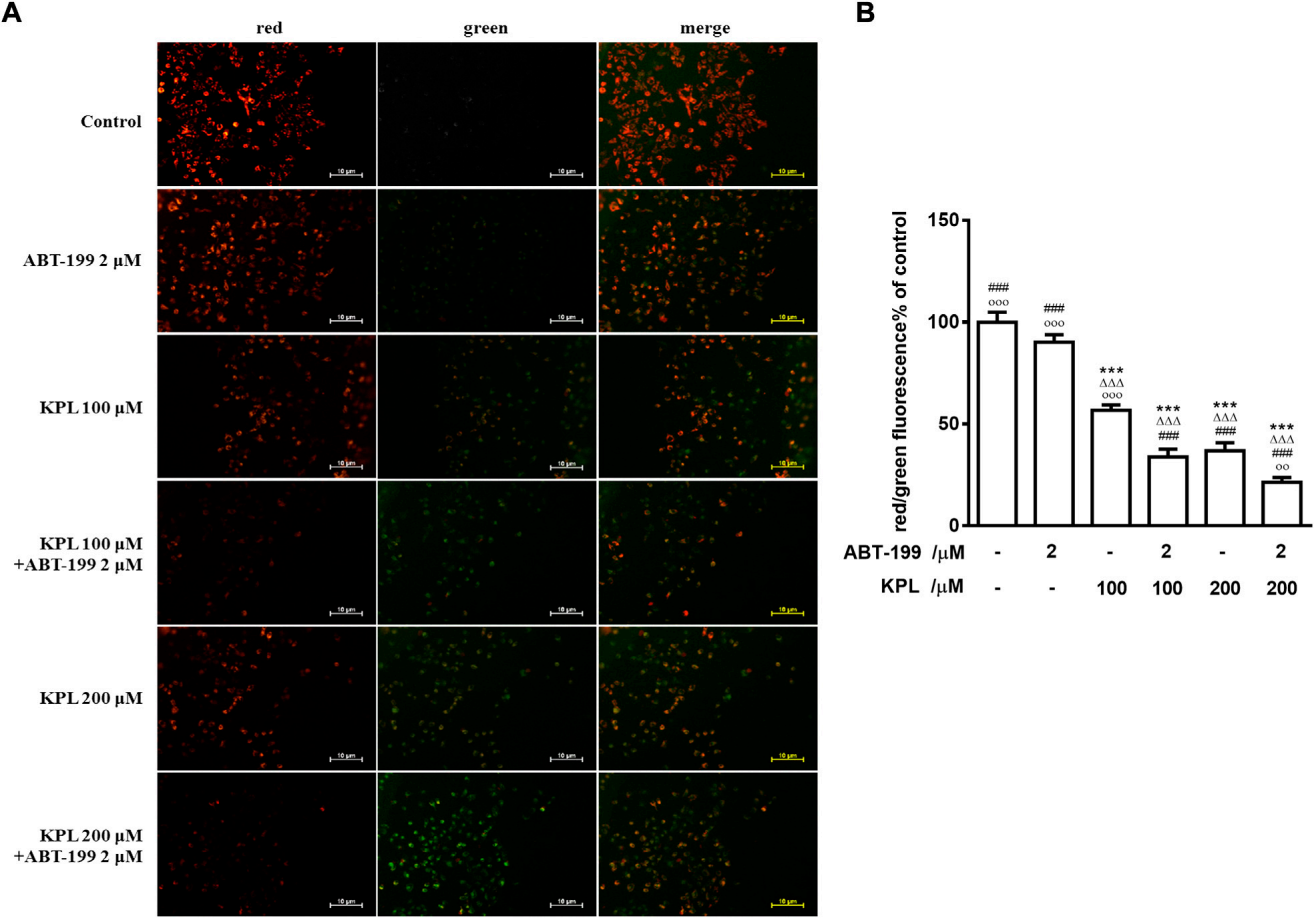
The effect of KPL combined with ABT-199 on mitochondrial membrane potential of HepG2 cells. **(A)** Representative images of double immunofluorescent staining for JC-1. Different concentrations of KPL with or without 2 μM ABT-199 were treated on HepG2 cells for 24 h, and then the changes of mitochondrial membrane potential were detected with JC-1 dye. The negative group was untreated. The images were collected by inverted fluorescence microscope. Magnification = 100 ×. **(B)** The ratio of red and green fluorescence intensity. Values are means ± SD (*n* = 12). ***p < 0.001 *versus* negative control group; ^ΔΔΔ^ *p*<0.001 *versus* ABT-199 alone group; ^###^ *p* < 0.001 *versus* KPL 100 μM group; ^○○^ *p* < 0.01, ^○○○^ *p* < 0.001 *versus* KPL 200 μM group.

One of the significant signs of early-stage apoptosis is the decrease of mitochondrial membrane potential (Elmore, 2007). In this study, JC-1 fluorescent probe was used to detect the changes of mitochondrial membrane potential after KPL combined with ABT-199 treatment of HepG2 cells. JC-1 staining results were shown in Figures 5A,B. The effect of cells treated with ABT-199 alone was not significantly different from the negative control group, which demonstrated bright red fluorescence. The weak green fluorescence in these cells indicated that JC-1 existed in the aggregated form in mitochondria and with high mitochondrial membrane. However, in KPL alone (100 μM and 200 μM) treatment group and KPL combined with the ABT-199 treatment group, the red fluorescence intensity in mitochondria decreased significantly and the green fluorescence in cytoplasm increased significantly. These findings indicated that JC-1 in mitochondria existed in the form of monomer with low mitochondrial membrane potential. The results showed that ABT-199 alone could not reduce the mitochondrial membrane potential of HepG2 cells, but the mitochondrial membrane potential decreased significantly after being combined with KPL.

### 3.7 The effects of KPL combined with ABT-199 on endogenous mitochondrial apoptosis pathway-related proteins in HepG2 cells

**FIGURE 6:**
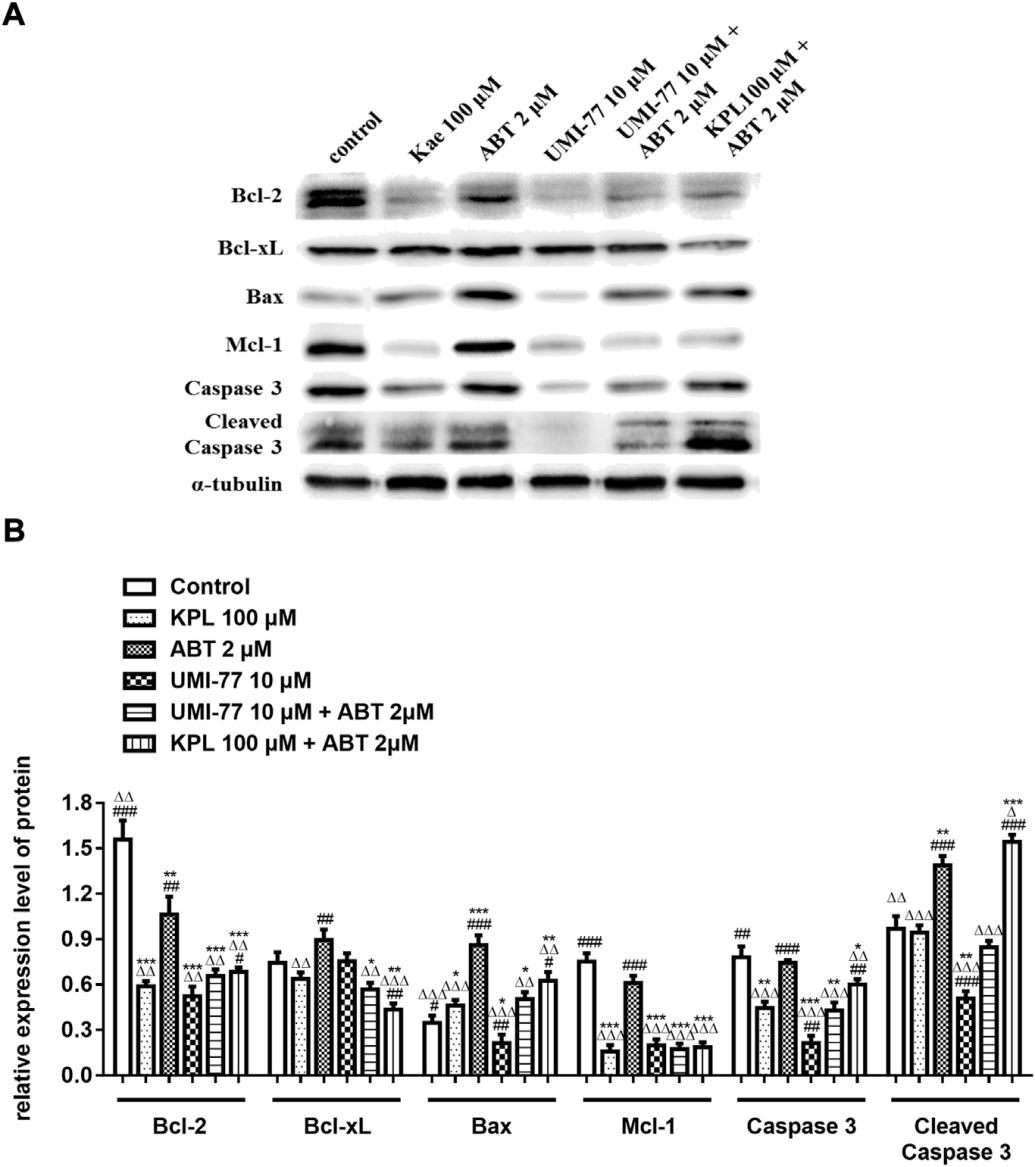
The effects of KPL combined with ABT-199 on caspase-3, cleaved caspase-3, Bcl-2, Bcl-xL, Bax and Mcl-1 proteins in HepG2 cells. **(A)** The expression levels of Bcl-2, Bcl-xL, Bax, Mcl-1, caspase 3 and cleaved caspase 3 were examined by western blotting analysis. α-tubulin was used as internal control. **(B)** The relative expression level of Bcl-2, Bcl-xL, Bax, Mcl-1, caspase-3 and cleaved caspase-3. α-tubulin was used as internal control. Relative expression of target protein = expression of target protein/expression of internal control protein. Values are means ± SD (*n* = 3). *p < 0.05, **p < 0.01, ***p < 0.001 *versus* negative control group; ^ΔΔΔ^ *p*<0.001 *versus* ABT-199 alone group; ^###^ *p* < 0.001 *versus* KPL 100 μM group.

**FIGURE 7:**
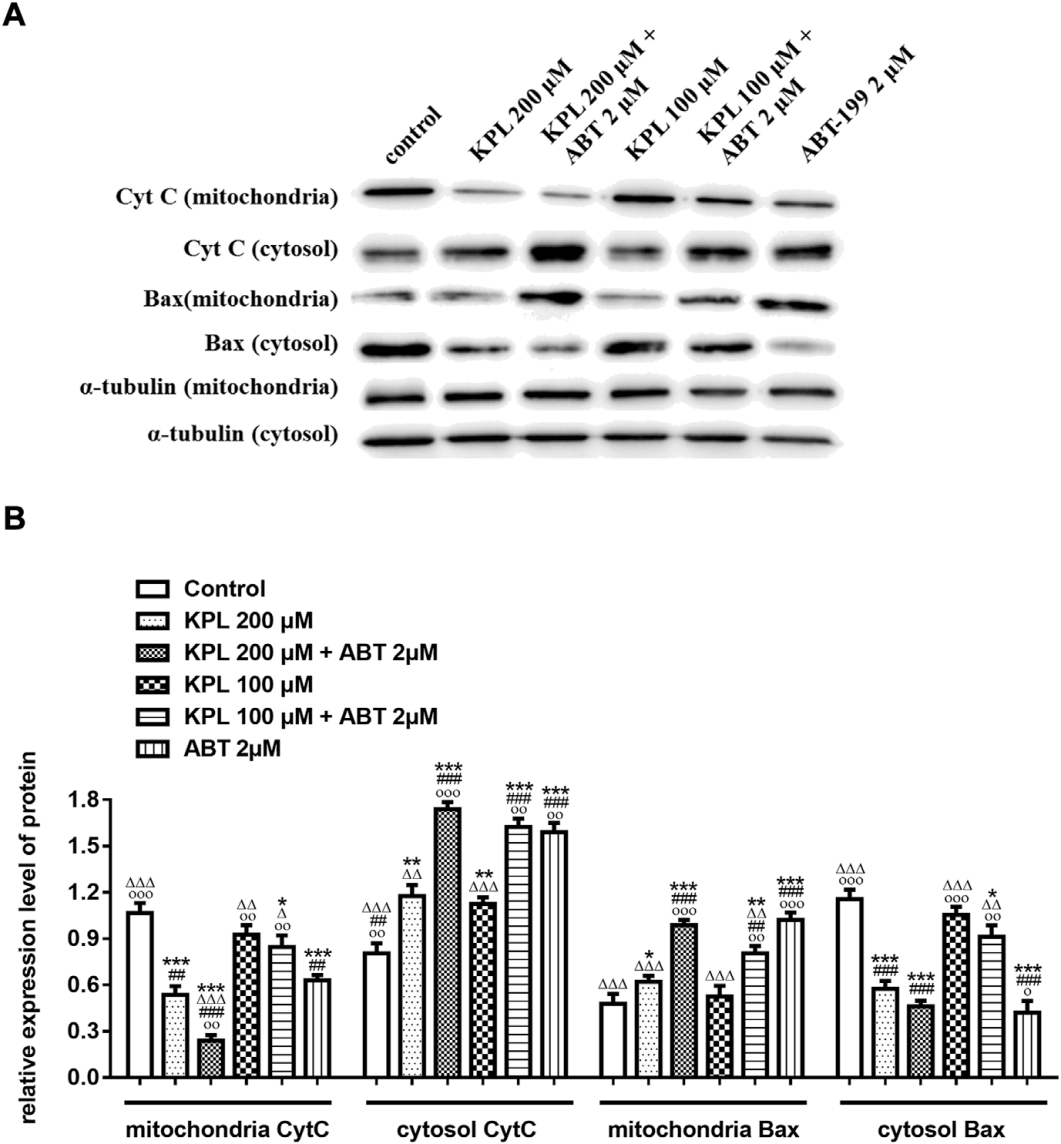
The effects of KPL combined with ABT-199 on cytochrome C release and Bax translocation in HepG2 cells were detected in mitochondria and cytoplasmic proteins by Western blot assay. **(A)** The expression levels of cytochrome C release and Bax in mitochondria and cytoplasmic were examined by western blotting analysis. α-tubulin was used as the internal control. **(B)** The relative expression levels of cytochrome C release and Bax in the mitochondria and the cytoplasm. α-tubulin was used as the internal control for relative expression of target.

To further explore the molecular mechanism of KPL combined with ABT-199 against HCC, we have proceeded with the Western Blot technique. In this experiment, Mcl-1 inhibitor UMI-77 was used as the positive control. As shown in Figures 6A,B, when the HepG2 cells were treated with ABT-199 alone, there was no significant difference in the expression level of anti-apoptotic protein Mcl-1 compared with the negative control group; ABT-199 combined with UMI-77, an Mcl-1 inhibitor, also showed no significant difference in the expression of Mcl-1 compared with UMI-77 alone. The above results showed that ABT-199 could not directly inhibit the expression of Mcl-1. KPL alone and combined with ABT-199 could significantly inhibit the expression level of anti-apoptotic protein Mcl-1 (*p* < 0.001), and the inhibition intensity was similar to that of UMI-77 alone and UMI-77 combined with ABT-199. The findings from the present study also indicated that ABT-199 alone was insensitive to Mcl-1 and Bcl-xL in HepG2 cells. Interestingly, ABT-199 combined with KPL, compared with the negative control group, the expression of Bcl-2, including Mcl-1 and Bcl-xL, had decreased significantly (*p* < 0.001), indicating that KPL can enhance the inhibitory effect of ABT-199 on Bcl-2, Mcl-1 and Bcl-xL expression. It can also be observed from Figure 6 that compared with the negative control group, ABT-199 alone can significantly enhance the expression of Bax (*p* < 0.001), and even significantly greater than in ABT-199 combined with KPL. As reported in Figure 6, compared with the negative control group, there was no significant difference in the expression of caspase-3 when ABT-199 was applied alone (*p* > 0.05), but the expression of caspase-3 in other groups decreased significantly. Furthermore, as compared with the negative control group, KPL alone and UMI-77 combined with ABT-199 had no significant effect on the expression of cleaved caspase-3 after activation (*p* > 0.05), but ABT-199 alone and ABT-199 combined with KPL significantly increased the expression of cleaved caspase-3 (*p* < 0.01 or *p* < 0.001). ABT-199, as a selective Bcl-2 inhibitor, can cause apoptosis by interfering with the interaction among Bcl-2 proteins, subsequently promote Bax from cytoplasm to mitochondria, and mediate the release of cytochrome C from the mitochondria to cytoplasm. As shown in Figures 7A,B, in KPL alone (100 μM and 200 μM) treatment groups and KPL combined with ABT-199 treatment group, cytochrome C was released into the cytoplasm, therefore the expression of cytochrome C in mitochondria was reduced. It is interesting to note that the expression of cytochrome C in mitochondria of the combined treatment was less than that of KPL alone treatment, indicating that combined treatment can promote the release of cytochrome C into the cytoplasm. In addition, after treatment, the pro-apoptotic protein Bax was translocated to mitochondria, and its expression in mitochondria increased, and the expression of Bax of combined treatment was more than that of KPL only treatment. Western blot results showed that the inhibitory effect of KPL combined with ABT-199 on the proliferation of hepatoma HepG2 cells may be related to KPL downregulating Mcl-1, thereby regulating the Bcl-2 protein family in the endogenous mitochondrial pathway of ABT-199 regulating apoptosis.

## 4 Discussion

In this study, we first evaluated the sensitization effects of KPL on HepG2 hepatoma cells for ABT-199. The results of CCK-8 method and colony formation assay indicated that treatment with ABT-199 alone did not affect the hepatoma cells, but in combination with KPL, it could significantly inhibit the proliferation and the colony forming ability of HepG2 cells. To further verify the sensitization effect of KPL on ABT-199 *in vivo*, the H22 liver cancer bearing mouse model was employed, evaluated *via* the weight and pathological sections of tumour tissue.

Apoptosis is an important biological phenomenon of eukaryotic cells. In tumour suppression therapy, inducing tumour cell apoptosis is an important therapeutic strategy. To study the mechanism of KPL on ABT-199-sensitized hepatocellular carcinoma, Hoechst 33342 staining and JC-1 staining were used to observe the effect of KPL combined with ABT-199 on apoptosis of HepG2 cells. The results of apoptosis showed that KPL combined with ABT-199 could promote the early and late apoptosis of HepG2 cells, suggesting that the inhibitory effect of KPL combined with ABT-199 on cell proliferation was related to the induction of apoptosis. It was found that the apoptosis of different cell lines *via* varied factors was accompanied by the decline of mitochondrial membrane potential. The mitochondrial membrane potential began to decline before the pathological changes in the early stage of apoptosis, even earlier than DNA fragmentation (Salvioli et al., 2000). In this study, the JC-1 staining results showed that in the groups of combined medication, the red fluorescence intensity in mitochondria decreased significantly and the green fluorescence in cytoplasm increased significantly, indicating that JC-1 in mitochondria existed in the form of monomer, and the mitochondrial membrane potential was low (Zhao et al., 2018), demonstrating that the role of KPL in enhancing ABT-199 on inhibiting HepG2 cell proliferation and promoting apoptosis was related to the endogenous mitochondrial pathway.

When the cells were injured, the mitochondrial membrane potential decreased, and the membrane permeability increased. A series of apoptotic factors will be released, resulting in apoptosis. Bcl-2 family proteins are important proteins in the regulation of the apoptosis signaling pathway (REF), including the mitochondrial-mediated endogenous apoptosis pathway. The functions of the anti-apoptotic protein Bcl-2 and the pro-apoptotic protein Bax are diametrically opposed: Bcl-2 is a mitochondrial outer membrane protein, which can inhibit the release of cytochrome C with a pro-apoptotic effect from mitochondria to cytoplasm and then inhibit apoptosis (Naseri et al., 2015); anti-apoptotic protein Mcl-1 blocks the release of cytochrome C from mitochondria by interacting with pro-apoptotic factors of Bcl-2 protein family, such as Bim and Bak, so as to prevent cells from entering the process of apoptosis (Chen and Fletcher, 2017). Bax is a nuclear coding protein existing in advanced eukaryotes, which can be translocated to the outer membrane of mitochondria to mediate cell apoptosis. Bax itself can form a homodimer or heterodimer with Bcl-2. When Bcl-2 was highly expressed in cells, the heterodimer of Bcl-2 and Bax increased and the trend of apoptosis decreased; when Bax is highly expressed in cells, the homodimer formed by Bax itself is dominant, and it antagonizes the effect of Bcl-2, it will be prone to apoptosis (Oltvai et al., 1993; Naseri et al., 2015; Guo et al., 2018). Caspase is a cysteine aspartate specific protease that exists in the cytoplasm and plays an extremely key role in the process of apoptosis. Caspase-3 is located downstream of the cascade reaction. As a protein that cuts the cell structure, caspase-3 can directly cause cell apoptosis (Kirsch et al., 1999; Pisani et al., 2020). Caspase-3 activation is an important indicator for determining the state of cell apoptosis (Tyas et al., 2000; Brentnall et al., 2013; Nichani et al., 2020). When cells are stimulated by apoptosis signals or other harmful substances, caspase-3 will be activated into an active cleaved caspase-3. Cleaved caspase-3 participates in the process of cell apoptosis by cutting specific substrates, DNA dependent protein kinases, actin, lamin, *etc.* Therefore, the high expression of cleaved caspase-3 can induce tumour cell apoptosis, and it is one of the key proteins that promote cell apoptosis (Hou et al., 2015; Manning and Toker, 2017).

**FIGURE 8:**
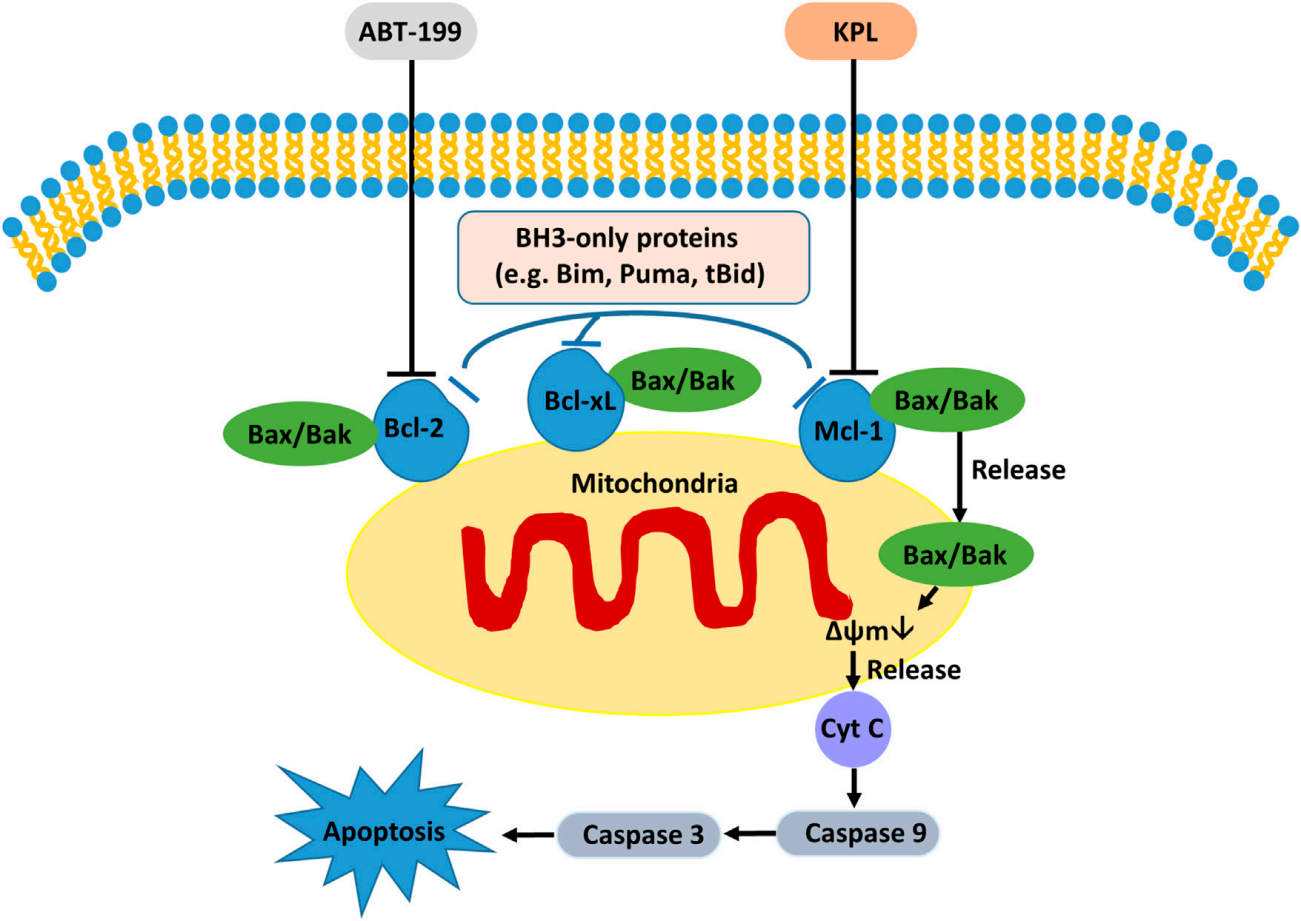
The possible molecular mechanism of KPL sensitizing ABT-199 against the proliferation of HepG2 cells.

Studies have shown that Mcl-1, an important anti-apoptotic protein in the mitochondrial apoptotic pathway, is overexpressed in liver cancer cells and liver cancer stem cells (Zhang et al., 2019). The increased expression of Mcl-1 is also one of the potential mechanisms of ABT-199 resistance (Niu et al., 2016). Because of the binding of ABT-199 with Bcl-2, although it prevents the interaction between Bim and Bcl-2, free Bim will be bound by the anti-apoptotic protein Mcl-1, which then blocks the signal of apoptosis transmitted by Bim, thus hindering apoptosis (Niu et al., 2016; Conage-Pough and Boise, 2018). In the signal transduction of apoptosis, anti-apoptotic proteins Bcl-xL and Bcl-2 can be combined by apoptosis promoter protein Bid and factors with similar functions to cause cytochrome C outflow (Shamas-Din et al., 2013), which further activates caspase-3 and caspase-7 to execute apoptosis (Galluzzi et al., 2012). Bcl-xL is a protein encoded by Bcl-2-like 1 gene and a major member of the Bcl-2 family. In terms of anti-apoptosis, Bcl-xL is more potent than Bcl-2 and Mcl-1 (Fiebig et al., 2006; Chen et al., 2015), and overexpression of Bcl-xL can reduce apoptosis in cells (Fuchsluger et al., 2011). Therefore, the high expression of Bcl-xL in tumour cells suggests that it is related to tumour chemoresistance (Liu et al., 1999). ABT-199 has a strong inhibitory effect on Bcl-2 and can promote the apoptosis of tumour cells with high Bcl-2 expression. However, ABT-199 as a BH3-mimetic has a weak inhibitory effect on Mcl-1, Bcl-xL and Bcl-w (Cang et al., 2015; Luedtke et al., 2017), our experiment results also proved it. The western blot results indicated that KPL could downregulate the expression of Bcl-2, Mcl-1 and caspase 3, and upregulate the expression of Bax. On the other hand, ABT-199 could downregulate the expression of Bcl-2 and upregulate the expression of Bax and cleaved caspase-3. When the HepG2 cells were co-treated with KPL and ABT-199, the expression of Bcl-2, Mcl-1 and Bcl-xL were significantly downregulated, and the expression of Bax, caspase-3 and cleaved caspase-3 were upregulated. But from Figure 6 we can see that compared with the negative control group, ABT-199 alone can significantly enhance the expression of Bax, and even significantly greater than in ABT-199 combined with KPL. This may be related to ABT-199’s ability to prevent the anti-apoptotic Bcl-2 from binding to pro-apoptotic Bax and Bak-1 proteins (Bordeleau et al., 2018). This also shows that, although ABT-199 can up-regulate the expression of Bax protein, the inhibition of ABT-199 on Mcl-1, Bcl-xL and Bcl-w is very weak, so from our experimental results, we can see that ABT-199 alone has little effect on the apoptosis of HepG2 cells with high expression of Mcl-1. KPL combined with ABT-199 could inhibit the expression of Bcl-2, Bcl-xL and Mcl-1, change the conformation of Bax and move to the surface of mitochondria, form pores on the mitochondrial membrane, and then reduce the mitochondrial membrane potential, release the apoptotic factor cytochrome C to the cytoplasm, trigger the cascade reaction of the caspase family, activate the effector caspase 3, and lead to apoptosis. Therefore, KPL combined with ABT-199 had a mutual sensitization effect on the Bcl-2 proteins family in the endogenous mitochondrial pathway of apoptosis, thus inducing HepG2 hepatoma cells apoptosis. The molecular mechanism of KPL sensitising ABT-199 against HepG2 cells is shown in Figure 8.

## 5 Conclusion

In conclusion, KPL can enhance the sensitivity of ABT-199 to hepatoma cells, inhibit the proliferation of HepG2 cells and induce apoptosis. The anti-proliferation effect of KPL combined with ABT-199 may be related to the down-regulation of Mcl-1 by KPL, which enhances ABT-199 regulates the Bcl-2 protein family in the endogenous mitochondrial pathway of apoptosis. Therefore, KPL combined with ABT -199 has a potential application prospect in the treatment of hepatocellular carcinoma, and can be used as a new strategy for the treatment of hepatocellular carcinoma patients. At present, Mcl-1 expression is mainly through the activation of Janus kinase/signal transducer and activator of transcription (JAK/STAT) signal pathway, phosphatidylinositol 3-kinase (PI-3K) signal pathway, mitogen activated protein kinase (MAPK) signaling pathway plays a role (Booy et al., 2011; Chou et al., 2013). The present study has yet to examine the signaling pathway of KPL sensitized ABT-199 in down-regulating Mcl-1, which may be an interesting research topic in the future. Furthermore, similar pharmacological studies on ABT-199 and KPL combination therapy in other HCC cell lines should be conducted in the future to augment the findings in the current study, in order to provide a stronger basis for the translational potential in clinical setting.

## Data Availability

The raw data supporting the conclusion of this article will be made available by the authors, without undue reservation.
